# Feasibility and acceptability of an innovative adherence intervention for young adults with childhood-onset systemic Lupus Erythematosus

**DOI:** 10.1186/s12969-020-00430-z

**Published:** 2020-04-26

**Authors:** Onengiya Harry, Lori E. Crosby, Constance Mara, Tracy V. Ting, Jennifer L. Huggins, Avani C. Modi

**Affiliations:** 1 Division of Rheumatology, Cincinnati Children’s Hospital Medical Center, Cincinnati, OH USA; 2 Section of Pediatric Rheumatology, Department of Pediatrics, Brenner Children’s Wake Forest Baptist Health, Medical Center Boulevard, Winston Salem, NC USA; 3 Division of Behavior Medicine and Clinical Psychology, Cincinnati Children’s Hospital Medical Center, Cincinnati, OH USA; 4 College of Medicine, University of Cincinnati, Cincinnati, OH USA

**Keywords:** Adherence intervention and young adults with cSLE, Adherence in cSLE, Adherence in pediatric-onset lupus, Adherence in cSLE

## Abstract

**Background:**

In Childhood-Onset Systemic Lupus Erythematosus (cSLE), poor medication adherence rates are very high. Interventions targeting this problem in cSLE are limited thus effective interventions are needed. The objective of this study is to examine the feasibility and acceptability an intervention (automated digital reminders + personalized prescribed treatment plan (pPTP)) to improve medication adherence in young adults with cSLE over 3 months.

**Method:**

This is a proof-of-concept randomized controlled study. All participants received SimpleMed+ pillboxes that track adherence. The treatment group received a pPTP, and in month 2, preselected digital reminders for missed doses. Reminders were discontinued after 30 days and adherence data collected. Data analysis was done using t-tests.

**Results:**

Twenty-one participants were approached and nineteen consented to participate, yielding a recruitment rate of 86%. Participants were on average 20.5 years, mostly black (58%) and female (84%). Of the nineteen consented, eleven were randomized to control (57%) and eight to treatment (42%) groups respectively. All participants in the treatment group rated the pillbox as easy to use, notably; none reported boredom with the pillbox or reminders. Also, 88% of participants in the treatment group rated the pillbox as helpful, however, only 50% reported the pPTP taught them new information about lupus or made them more interested in their lupus management.

**Conclusions:**

This is the first use of an electronic pillbox to track adherence to multiple medications in cSLE. The high rating of the pillbox makes it an acceptable method of measuring adherence. Feasibility and acceptability ratings for the intervention were mixed suggesting a there is a subset of cSLE patients for whom this intervention would be beneficial. Future research should focus on a larger trial.

## Background

In Childhood-Onset Systemic Lupus Erythematosus (cSLE), only 50–60% of patients take medications as prescribed [1]. Poor medication adherence (i.e. the extent to which an individual’s behavior matches treatment regimens) contributes to the morbidity and mortality [2] experienced by those diagnosed with this chronic, autoimmune, multisystem, inflammatory condition. Unfortunately, poor adherence is associated with lower health-related quality of life, increased health care utilization, health care costs, and unnecessary medication changes [3–5]. Optimizing medication adherence in individuals with cSLE is critical because, compared with adult-onset SLE patients, they have a more severe and prolonged disease course with more disease-related organ damage [6].

Characteristics unique to cSLE make adherence particularly difficult, including number of medications, the complexity of medication regimens, medication-related organ damage, disease flares, and psychological manifestations [7, 8]. For young adults with rheumatic diseases, adherence barriers include forgetfulness, polypharmacy, cost, personal problems, and/or refusal [9, 10]. Specific to those with cSLE, medication-specific knowledge deficits and the lack of perceived improvement in daily symptoms are significant barriers to medication adherence [2, 10, 11]. In addition, real or perceived side effects, low medication literacy, and/or poor medication taste were identified as adherence barriers in cSLE. Unfortunately, adherence barriers are stable and unlikely to improve without interventions [12, 13].

Despite the need for adherence interventions in adolescents and young adults with cSLE, few exist. Ting et al. developed a cellular text messaging intervention to remind adolescents and young adults with cSLE to take their medications but found reminders alone ineffective [14]. In contrast, multicomponent interventions that include educational, behavioral, and/or organizational strategies produce medium effects to improve adherence [15, 16]. A recent study by Scalzi and colleagues supports the use of multicomponent interventions in young adults with SLE [1]. In their study, participants were assigned to either a web-based educational module or a web-based educational module and social media intervention. Participants in the latter group showed significant improvement in medication adherence.

Our team developed a multicomponent adherence intervention targeting key adherence barriers identified by those with cSLE: medication-specific knowledge (i.e. goal of medication, regimen, side effects, and monitoring parameters) and forgetting. Personalized prescribed treatment plans have been found to improve disease-specific knowledge in pediatric chronic conditions and subsequent adherence behaviors [17]. Automated digital reminders target the barrier of forgetting by reminding patients to take their medications at specified times using mobile devices and/or compartment lights and audible alarms [18]. Automated digital reminders have been show to significantly improve short term medication adherence in adult populations [19], but need to be further studied in adolescents and young adults whose information consumption patterns best align with technology use [20].

The primary objective of this proof of concept randomized controlled study was to assess the feasibility and acceptability of a multicomponent intervention focused on improving medication adherence in young adults with cSLE. The intervention included two primary components: a prescribed treatment plan to ensure patients were knowledgeable about their treatment regimen and automated digital reminders to help them remember to take their medications. We hypothesized that (1) participants would rate the intervention as easy to use, acceptable, and beneficial, and (2) medication adherence would improve in the treatment group (prescribed treatment plan plus digital reminders) from pre- to post-intervention and be sustained 1 month after completing the intervention compared to treatment as usual (control group).

## Methods

### Study design and procedures

Participants were randomized to one of two groups (i.e., control versus treatment). To minimize bias in the randomization process, a computer-generated table generated by the study’s biostatistician determined the randomization scheme. Adherence was monitored by an electronic pillbox for both groups. While the control group received treatment as usual, the treatment group received a personalized prescribed treatment plan (pPTP) and automated digital reminders for 1 month.

At enrollment, participants (young adults) completed a battery of questionnaires and were given an electronic adherence pillbox (SimpleMed+, Vaica), which was used for the entire 3-month study period. A medical chart review was conducted at enrollment. At the end of the study, participants in the treatment group completed a feasibility and acceptability questionnaire within 2 weeks.

### Participants and recruitment

Participants were recruited during routine pediatric rheumatology clinic visits at a Midwestern children’s hospital. Inclusion/exclusion criteria were: 1) confirmed diagnosis of cSLE via chart or registry review, 2) patient between ages 18 and 24 years 3) English-speaking, and 4) no significant developmental delay or cognitive dysfunction (i.e. autism spectrum disorder). Eligible participants were approached by study staff at regularly scheduled clinic. All questions were addressed, and written informed consent was obtained. This study protocol was approved by the hospital’s Institutional Review Board.

### Measures

#### Demographic information

A demographic information questionnaire completed by young adults included patient’s age, race, ethnicity, time to diagnosis, and year of diagnosis. Information regarding cSLE diagnosis, presence of lupus nephritis, SLE Disease Activity Index (SLEDAI), treatment regimen, and medical and psychosocial co-morbidities were abstracted from the electronic medical record (EMR).

#### Barriers to Adherence Tool (BAT) [9]

This fourteen item checklist assesses logistical, social, psychological and knowledge-based adherence barriers in patients for all treatment modalities (medications, injections, infusions and physical/occupational therapy) and was administered at baseline. While validation of this tool has not been done in cSLE, the themes represented by this measure have been scientifically validated in other chronic pediatric conditions [21].

#### Pain intensity and fatigue

Participants completed both the PROMIS® Pain Numeric Rating Scale v.1.0 (Pain Intensity 1a) and PROMIS Fatigue Short Form v1.0 (Fatigue 4a) at baseline to ensure no differences between the control and treatment groups. The Patient-Reported Outcomes Measurement Information System (PROMIS®) is a set of validated, person-centered measures that standardize patient reported outcome assessment for use in both research and health care settings [18]. PROMIS® consists of item banks with variable number of questions that can be combined to form multi-item measures of varying length and complexity [18, 19]. Both measures employ a Likert-type scale. For pain intensity, a higher score denotes greater pain. For the PROMIS® fatigue, a T-score is calculated in which 50 is the mean of a relevant reference population and 10 is the standard deviation of that population [18, 20]. Higher T scores represent greater fatigue.

### Feasibility and acceptability

This was assessed using: 1) enrollment and retention rates, 2) ability to utilize and return the electronic adherence monitoring device, 3) proportion of completed questionnaires, and 4) acceptability of the automated digital reminder and pPTP intervention as characterized by a written questionnaire.

#### Feasibility and acceptability questionnaire

This study-specific survey assessed feasibility and acceptability of the adherence intervention. The survey contains 12-items including information on ease of use, psychological benefits, and new knowledge about lupus, using a Likert-scale with scores ranging from 1 to 5. A score of 4 and 5 indicated agreement or strong agreement with intervention features (e.g. ease of using pillbox or digital reminders). Similar questionnaires have been used by adherence researchers and provide critical information on ways to improve adherence interventions [22].

### Adherence outcomes

#### Medication adherence self report inventory (MASRI)

The MASRI is a 6-item questionnaire of self-reported medication adherence assessing the frequency of medication intake and has been validated in patients with lupus [23, 24]. Only the visual analog scale (VAS) item of the MASRI was used to determine a numeric estimate of adherence (0–100%). This VAS has acceptable internal consistency (Cronbach’s α = 0.7) and good reliability [24].

#### Objective adherence

All study participants were asked to use a SimpleMed+ pillbox to organize and administer their medications. The SimpleMed+ pillbox measures 17 cm (width) × 30.4 cm (diameter) × 3.5 cm (height), with 28 compartments, enough to store up to four doses of multiple medications for each day of the week. This equipment has a self-contained cellular modem, on-board reminders (compartment lights or sounds) and additional reminders (text messages) [18]. When a compartment is opened, a date/time stamp is sent wirelessly to a report that can be accessed on Vaica Medical’s secure website.

### Study interventions

#### Usual care

Participants received a standard after visit summary provided by their provider. Any questions regarding their visit plan or prescribed medications were addressed by their provider or nursing staff per customary clinic practice.

#### Treatment

##### Personalized prescribed treatment plan

Participants in the treatment group received a tool designed by the authors to provide clear information regarding the individual’s treatment plan in conjunction with their clinic after visit summary from the electronic medical record. Prior to use in study, feedback on the prescribed treatment plan was obtained from young adults with cSLE regarding clarity and content. This instrument contains information about medication by functional classification (i.e. *core medication = hydroxychloroquine, fast-acting immune suppressant = > steroids,* etc.*)*. The prescribed treatment plan also includes an “*other*” category which covers vaccines, supplements, contraceptives, information on adverse effects and monitoring parameters for each class. Participants were oriented to the treatment plan and all of their medications were documented (including dosage and timing). All questions regarding the prescribed treatment plan were answered by the first author.

##### Automated digital reminders

The treatment group also received automated digital reminders for 30 days beginning in month 2 of the study. Participants could select up to 3 automated digital reminders (i.e. text messages, compartment lights, or sound); all but one participant chose text messages. During the active treatment period (month 2), automated digital reminders began for the treatment group while control participants continued to use their electronic monitors without receiving reminders. At the beginning of month 3, the automatic digital reminders were turned off for the treatment group. Study personnel accessed Vaica Medical’s secure website three times a week to record logged adherence information.

### Statistical analysis

Data analytic procedures were carried out using SAS version 9.4 (SAS Institute Inc., Cary, NC). Data were entered and cross-checked for accuracy. Descriptive statistics (e.g. frequency, mean, range, SD) were calculated for demographic, disease severity, self-reported adherence, and feasibility/acceptability variables. Two sample independent t-tests were used to examine differences between control and treatment groups at baseline.

Adherence was calculated as the number of doses taken/number of doses prescribed *100% as measured by the adherence monitors. Adherence ranged from 0 to 100% for all analyses [22, 25]. A power analysis was not calculated as the primary aims of this study were feasibility and acceptability of this intervention.

## Results

### Baseline demographic and disease parameters

Demographical and disease characteristics are summarized in Table 1. Overall, participants were on average 20.5 years (32% White; 58% Black; 1% Asian; 1% multiple races; 84% female; and 16% male). All participants had at least four of the 11 ACR classification criteria for SLE at time of diagnosis. Forgetting to take medication, medication side effects, fertility concerns, and taste of medication were top barriers to medication adherence (see Table 1). At baseline, control and treatment groups were similar except the control group had more female participants (*p* < 0.03), more fatigue (*p* < 0.001), and endorsed forgetting to take medications (*p* = 0.01) as a barrier to medication adherence more often. There was no significant difference in self-report of adherence (MASRI adherence score, *p* = 0.73), proportion with organ damage (*p* = 0.38), or pain intensity (*p* = 0.25).

**Table 1:** Baseline demographical, disease, and barriers characteristics of participants stratified by group

Characteristic	Total Participants (*n* = 19)	Control (*n* = 11)	Treatment (*n* = 8)
**Demographics**			
Age, mean, (SD) in yrs.	20.5 (1.6)	20.5 (1.6)	20.5 (1.7)
Gender (female), No (%)	16 (84)	11 (100)	5 (63)
Race, No, (%)			
White	6 (32)	4 (36)	2 (25)
Black	11 (58)	6 (55)	5 (63)
Asian	1 (5)	–	1 (13)
Multiple	1 (5)	1 (9)	–
Insurance type, No (%)			
Public	7 (37)	5 (45)	2 (25)
Private	12 (63)	6 (55)	6 (75)
**cSLE**			
Disease duration, yrs., (SD)	4.8 (3.2)	4 (3)	5.4 (3.4)
SLEDAI^a^, mean, (range)	5 (0–10)	5 (2–10)	4.4 (0–9)
SLICC^b^, No., (%)	4 (40)	3 (30)	1 (12.5)
Physician global, mean (range)	0.9 (0–3.5)	1 (0–3.5)	0.6 (0–1.5)
Nephritis, No. (%)	8 (42)	5 (45)	3 (38)
PROMIS fatigue, mean (SD)	53 ± 3.0	57 ± 2.9	47 ± 3.2
PROMIS pain intensity, mean (SD)	2.8 (2.3)	3.6 ± 2.3	1.8 (1.9)
No of Pills^c^, mean (SD)	7 (3.8)	7 (3.5)	8 (4.2)
Presence of comorbidity (%)	18 (95)	11 (100)	7 (88)
Hypertension, No. (%)	5 (26)	3 (27)	2 (25)
Depression, No. (%)	5 (26)	3 (27)	2 (25)
Obesity, No. (%)	10 (53)	7 (66)	3 (38)
Barriers^d^, No (%)			
Forgetting	15 (79)	11 (100)	4 (50)
Taste	13 (68)	9 (81)	4 (50)
Side effects			
Current	12 (63)	8 (73)	4 (50)
Future	11 (58)	6 (55)	5 (63)
Fertility concerns	11 (58)	7 (64)	4 (50)
Treatment upsetting	8 (42)	6 (55)	2 (25)
Inconvenient	7 (37)	6 (55)	1 (13)
Cost	7 (37)	5 (45)	2 (25)

^a^SLEDAI 2 K score from clinic visit preceding start of study

^b^SLICC Damage Index obtained from annual calculated score within 12 months of study initiation

^c^Includes both lupus and non-lupus medications being taken by participants

^d^Includes barriers with fewer than 3 respondents such as difficulty swallowing pills, running out of medication, refusing to take treatment, believing treatment to be unnecessary, desire to keep treatment private from others, lack of perceived treatment benefit, treatment getting in the way, and hard instructions

### Feasibility and acceptability parameters

**Fig. 1:**
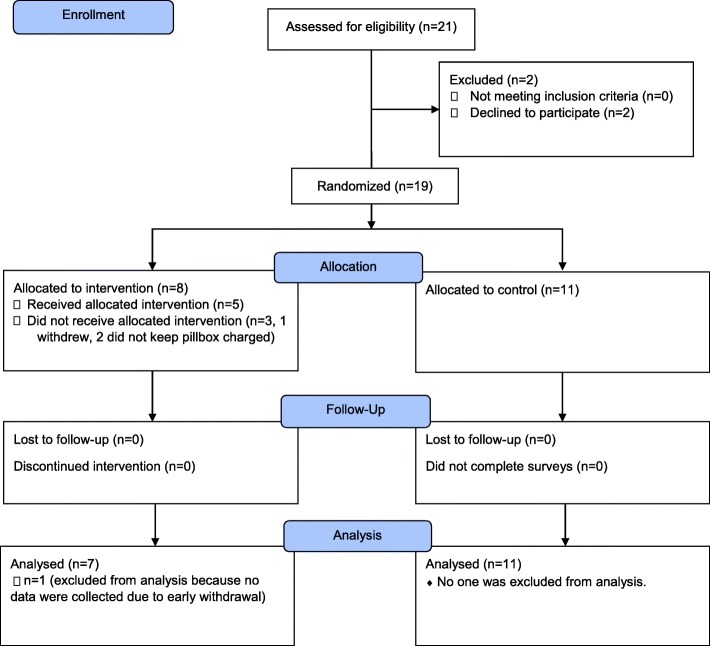
Consort Diagram

Twenty-one participants were approached for participation and nineteen consented to participate (see Fig. 1, Consort Diagram). Two individuals declined study participation yielding a recruitment rate of 86%. Reasons for non-participation were having only one medication daily and no desire to participate. Of the nineteen who consented, eleven were randomized to treatment as usual (57%) and eight were randomized to treatment (42%). Once allocated to groups, three participants in the treatment group did not receive the intervention (1 participant withdrew due to problems with equipment connectivity despite changing pillboxes and 2 participants did not keep the pillbox charged for monitoring). No participants were lost to follow up at study completion; however, the participant who withdrew was not included in objective adherence analyses.

Eighteen of nineteen participants completed all components of the study, used the pillbox, and returned it at study completion. Notably, all participants completed study questionnaires. Eighty-eight percent (7/8) of the treatment group actively used their SimpleMed+ pillbox. None of the participants in treatment group reported boredom with the pillbox or reminders. Responses on the feasibility and acceptability questionnaire are summarized in Table 2.

**Table 2:** Feasibility and acceptability results for the treatment group

Title Items	% Young Adult Who Agree or Strongly Agree^**a**^ (***N*** = 8)
1. The pillbox was easy to use.	100
2. The pillbox did not have any glitches.	75^b^
3. The pillbox was helpful.	88
4. The personalized treatment plan taught me something new.	50
5. The personalized treatment plan made me more interested in my lupus management.	50
	**% Young Adult Who Agree or Strongly Agree**^**a**^ **(*N*** **= 5)**
6. The pillbox plus digital reminders helped me take my lupus medicine.	60
7. I took my medication as soon as the reminders alerted me.	40
8. The reminders were useful to my lupus management.	60
9. I became bored with pillbox and reminders.	0
10. I will continue to use the pillbox with reminders in the future.	60
11. Overall, I benefited from using the pillbox with reminders.	60
12. I think the pillbox with reminders has helped reduce my stress about managing lupus.	60

^a^Based on a 4–5 score on a 5 point Likert Scale denoting agree or strongly agree. Assumption is made that a rating in this range notes a high rate of acceptability by respondents

^b^One participant had difficulty with pillbox cellular connectivity. The other participant chose a “neutral” response but had previously reported problems with not having electricity at home

### Objective adherence outcomes

**Fig. 2:**
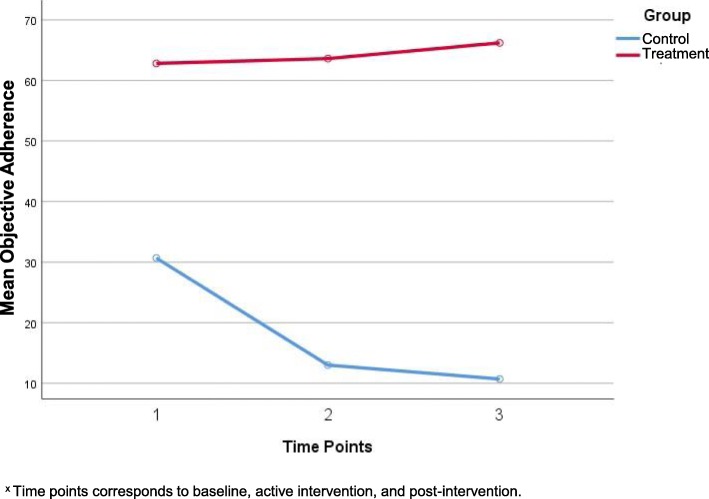
Means of Objective Adherence over Time

*Objective adherence.* Baseline objective adherence was different for both control and treatment groups despite randomization. The control group also had a steep decline in objective adherence that continued over the duration of the study (Fig. 2 –Means of Objective Adherence over Time). Notably, mean objective adherence for the treatment group continued to increase although this increase was not statistically significant (63 to 66, *p* = n.s.).

## Discussion

This proof-of-concept study of a multicomponent adherence intervention, focused on providing education about the individual’s treatment regimen and automated digital reminders to young adults with cSLE. Participants were able to use the pillbox and ensure cellular connectivity with minimal guidance. The high use and acceptability rating of the pillbox suggests that it a feasible and acceptable method of measuring objective adherence as an outcome measure for future trials. In contrast, acceptability ratings for the multi-component adherence intervention were mixed. While none of the participants became bored with the pillbox and reminders, only half of the responders rated the intervention highly favorable. Higher acceptability may have been reported by the 50% of participants who identified forgetting as a primary adherence barrier. This finding suggests that there is a subset of cSLE patients for whom an electronic pillbox with digital reminders would be beneficial. Half of the participants in the treatment group reported gaining new lupus-related knowledge and being more interested in the management of their lupus from the pPTP. This indicates that although education in general is helpful, educational interventions should be personalized to address specific knowledge deficits (i.e. side effects or fertility concerns). Overall, this multicomponent intervention warrants further study for the subset of patients for whom forgetting and certain medication-related knowledge deficits are significant barriers to medication adherence. Future studies should tailor interventions to more precisely target individual barriers to medication adherence.

Our objective adherence data suggests group differences in adherence, with the treatment group having higher adherence over time compared to the control group. Reasons for this group difference may be explained by expected reactivity to intervention. The effect of typical reactivity to the pillbox at baseline remains unclear for the treatment group. However, objective adherence for the control group shows a steep decrease from the first to second month suggesting a 4 week period of typical reactivity to the pillbox and/or intervention. This finding is consistent with adherence interventions in chronic illnesses [22, 25].

For the control group, objective adherence continued to decline over the duration of the study indicating that poor adherence to medication may not improve without intervention. A similar trend has been reported for those with poor medication adherence, without targeted intervention, in other chronic conditions [12, 26, 27].

While there were promising insights from this proof-of-concept study targeting a complex problem, we acknowledge a number of potential limitations. First, our sample size was small and thus we may have been underpowered to detect changes over time between groups. However, the main purpose of this study was to assess the feasibility and acceptability of using electronic pillboxes, provision of a personalized treatment plan and automated digital reminders. Second, this was a single site study that focused only on young adults, a high risk population for poor medication adherence, which may impact generalizability of results. Finally, although essential for unbiased group allocation, our randomization was not stratified to have equal groups due to the small sample size.

## Conclusion

Overall, our study indicates that electronic pillboxes are easy to use and may be used to track adherence in future studies, especially for those on multiple medications. Further research is needed to assess the correlation between this pillbox and other measures of adherence such as medication possession ratio or proportion days covered. Also, large-scale testing of the electronic pillbox with automated digital reminders as an adherence intervention for patients with cSLE is needed to detect effect sizes. If effective, this methodology has promise to increase medication adherence thereby decreasing negative health outcomes associated with poor adherence. Finally, continued clinical research that results in a refined approach to optimize the intervention by using the most effective components will be required to realize the full potential of electronic pillboxes for improving medication adherence in adolescents and young adults with cSLE.

## Data Availability

The datasets generated and/or analyzed during the current study are not publicly available due to the restrictions of the ethics approval originally obtained.

## Abbreviations

ACR: American College of Rheumatology
ANOVA: Analysis of Variance
BAT: Barriers to Adherence Tool
cSLE: Childhood-Onset Systemic Lupus Erythematosus
EMR: Electronic Medical Record
MASRI: Medication Adherence Self-Report Inventory
PROMIS®: Patient-Reported Outcomes Measurement Information System
SAS: Statistical Analysis System
SLE: Systemic Lupus Erythematosus
SLEDAI: SLE Disease Activity Index
pPTP: Personalized Prescribed Treatment Plan
VAS: Visual Analogue Scale
